# Randomised cluster trial to support informed parental decision-making for the MMR vaccine

**DOI:** 10.1186/1471-2458-11-475

**Published:** 2011-06-16

**Authors:** Cath Jackson, Francine M Cheater, Wendy Harrison, Rose Peacock, Hilary Bekker, Robert West, Brenda Leese

**Affiliations:** 1 School of Healthcare, University of Leeds, Leeds LS2 9UT, UK; 2 Institute for Applied Health Research, Glasgow Caledonian University, Glasgow, UK; 3 Leeds Institute of Genetics, Health and Therapeutics, University of Leeds, Leeds, UK; 4 Leeds Institute of Health Sciences, University of Leeds, Leeds, UK

## Abstract

**Background:**

In the UK public concern about the safety of the combined measles, mumps and rubella [MMR] vaccine continues to impact on MMR coverage. Whilst the sharp decline in uptake has begun to level out, first and second dose uptake rates remain short of that required for population immunity. Furthermore, international research consistently shows that some parents lack confidence in making a decision about MMR vaccination for their children. Together, this work suggests that effective interventions are required to support parents to make informed decisions about MMR.

This trial assessed the impact of a parent-centred, multi-component intervention (balanced information, group discussion, coaching exercise) on informed parental decision-making for MMR.

**Methods:**

This was a two arm, cluster randomised trial. One hundred and forty two UK parents of children eligible for MMR vaccination were recruited from six primary healthcare centres and six childcare organisations. The intervention arm received an MMR information leaflet and participated in the intervention (parent meeting). The control arm received the leaflet only. The primary outcome was decisional conflict. Secondary outcomes were actual and intended MMR choice, knowledge, attitude, concern and necessity beliefs about MMR and anxiety.

**Results:**

Decisional conflict decreased for both arms to a level where an 'effective' MMR decision could be made one-week (effect estimate = -0.54, p < 0.001) and three-months (effect estimate = -0.60, p < 0.001) post-intervention. There was no significant difference between arms (effect estimate = 0.07, p = 0.215). Heightened decisional conflict was evident for parents making the MMR decision for their first child (effect estimate = -0.25, p = 0.003), who were concerned (effect estimate = 0.07, p < 0.001), had less positive attitudes (effect estimate = -0.20, p < 0.001) yet stronger intentions (effect estimate = 0.09, p = 0.006). Significantly more parents in the intervention arm reported vaccinating their child (93% versus 73%, p = 0.04).

**Conclusions:**

Whilst both the leaflet and the parent meeting reduced parents' decisional conflict, the parent meeting appeared to enable parents to act upon their decision leading to vaccination uptake.

## Background

In the UK public concern about the safety of the combined measles, mumps and rubella [MMR] vaccine as a consequence of Wakefield et al's now discredited research continues to impact on MMR coverage [1] even though the sharp decline in uptake has begun to reverse [2]. Current first and second dose MMR uptake rates in England are 84% and 77% respectively [2], short of the 95% required for population immunity [3]. As a consequence, there is a pool of unimmunised children susceptible to the diseases, reflected in persistent localised measles outbreaks [4] with an epidemic predicted in the near future [5]. In some European countries and in the USA, childhood immunisation is mandatory yet MMR vaccine refusal has increased, similarly leading to measles outbreaks [6,7]. Repeated assertions by the Department of Health for England and Wales and the U.S Centers for Disease Control and Prevention that the MMR vaccine is safe have had limited effect on allaying parents' concerns in some sections of the community [8-10]. More than ten years after the publication of Andrew Wakefield's now discredited findings [11], there is some evidence that parent trust in MMR has improved [12], yet significant numbers continue to lack confidence in making an MMR decision [9,13-19] and many criticise what is perceived to be the poor quality of information provided [9,17].

A recent systematic review [20] identified the decision support needs of parents making child health decisions (including immunisation); these related to three themes: (i) a need for timely, consistent up-to-date evidence based information tailored to the individual child, delivered in a variety of formats from trustworthy sources; (ii) a need to talk with others facing the same decision to share experiences; and (iii) a need to be in control of their level of preferred involvement in the decision-making process. This suggests that interventions, informed by parents' expressed needs of what they would find helpful to make informed decisions about MMR are required. However, in spite of a large literature describing the factors that influence parents' decisions whether to vaccinate their child with MMR [21-25], evaluations of decision support interventions in this context are limited [26-29].

Informed by a systematic review [20] and an interview study with parents [9] we developed and evaluated an evidence-based, parent-centred, multi-component intervention to support informed parental decision-making for the MMR vaccine. This parent-centred approach is consistent with Western health policy in which a clinician-patient partnership is emphasised [30-32]. It is also in line with the fundamental tenets of health promotion [33] that is based on an 'engagement' model of communication where a key goal is empowerment. The intervention was designed to supplement routine UK primary care service for childhood vaccination whereby parents are invited to take their child, free of charge, for all immunisations on the National Health Service Routine Childhood Immunisation Schedule [34]. Childhood vaccination is not mandatory in the UK.

In this paper we report the findings of a cluster randomised controlled trial to evaluate a parent-centred, multi-component intervention to support informed decision-making for MMR [35]. The effectiveness data are presented here. Acceptability of the intervention is reported elsewhere [36].

## Methods

### Setting and Participants

The study was approved by the Local Research Ethics Committee (06/Q1107/25) on 18 May 2006. It was located in three of 33 wards (electoral district) in Leeds, England (approx. 770,000 population [37]). Using the Index of Multiple Deprivation [38] these wards were selected to represent low, medium and high deprived geographical areas [mean scores: low = 6.89; medium = 29.22; high = 55.07]. Primary healthcare centres employing at least two medical practitioners were purposively selected based on their low income scheme index [LISI, 39] scores. For example, in the high deprived ward, we approached centres with the most deprived practice population first (i.e. highest LISI score). Childcare organisations in the same wards were approached on the basis of size, the largest first. Eleven (of 15) healthcare centres and six (of eight) childcare organisations were invited to participate. Six primary care centres and six childcare organisations agreed.

Within these providers the target sample was parents who were English literate with a child eligible for the first or second dose MMR vaccination. At the time of the study, in the UK, the first dose was given at 13 months and the second dose between four and five and half years of age [34]. The target age range was, therefore, six months to five years. Letters were sent to eligible parents on providers' registers. Parents replied to the research team and were telephoned for screening and recruitment. Recruitment occurred May to July 2006.

### Design and Intervention

The design was a cluster randomised controlled trial design with two arms: intervention and control. This design was chosen to reduce the potential risk of contamination between arms. The six healthcare centres and six childcare organisations were matched in pairs based on their ward (three pairs of healthcare centres, three pairs of childcare organisations). One of each pair was randomly allocated to the intervention, the other to the control arm. A researcher not involved in the study and blind to the identity of clusters performed the randomisation using a sealed envelope procedure. The study researcher (RP) was blind to arm assignment when screening and recruiting parents, and sending out the baseline questionnaire. Statisticians (WH, RW) saw blinded data. Parents were blind to arm assignment at recruitment and in completing the baseline questionnaire.

Parents allocated to the intervention arm were invited to attend one two-hour parent meeting, co-facilitated by a researcher (CJ, FMC, RP) and a parent. Three parent facilitators (all women) were recruited from local communities. They received one half-day training. In advance of the meeting parents were sent an information leaflet ('MMR your questions answered', [40]). The content and delivery of the parent meeting (see Table 1) was informed by interviews with 69 parents [9] and a systematic review of parents' decision support needs [20]. This was refined in two focus groups with local parents. The meeting included three components: provision of balanced information, a group discussion and a coaching exercise [41].

**Table 1:** Overview of parent meeting

Time	Facilitator	Content	Aims
15 minutes	Parent facilitator and Researcher	**WELCOME** Introductions Outline aims of meeting Go through programme Agree ground rules for meeting	**Of meeting** To provide parents with the opportunity to discuss MMR with other parents who are making an MMR decision To provide information about MMR from a variety of perspectives To introduce and practice one approach to supporting parents to ask questions about MMR of their healthcare practitioner
30 minutes	Parent facilitator	**GROUP DISCUSSION** Aim of session Reminder of ground rules Introductory question - would anyone like to tell us what you hoped to get out of this parent meeting today? Discussion	**Of session** To provide parents with the opportunity to discuss any issues about MMR with other parents who are also making an MMR decision
30 minutes	Immunisation Nurse Specialist	**QUESTION AND ANSWER SESSION** Aim of session Parents ask questions of the immunisation nurse specialist Parents are alerted to resources that they can take away	**Of session** To provide parents with the opportunity to ask questions of the immunisation nurse specialist
35 minutes	Researcher	**COACHING EXERCISE** Aim of session Brief input on the importance of raising questions about MMR with a healthcare practitioner Introduce and discuss the question prompt sheet Role play exercise using the question prompt sheet Brief discussion on usefulness of the question prompt sheet and role play	**Of session** To introduce and practice one approach to supporting parents to ask questions about MMR in the primary care consultation
10 minutes	Parent facilitator and Researcher	**CLOSE OF MEETING** Thank parents and provide overview of next stage of research study	

Parents in the control arm were sent the same MMR leaflet.

### Measures

Parent characteristics (e.g. age, ethnicity) were collected by telephone at recruitment. Primary and secondary outcomes were collected by postal questionnaire prior to randomisation (T1), one week post-intervention (T2) and three months post-intervention (T3). The questionnaire was developed in collaboration with an expert in the field of health decision-making (HB) and piloted with five parents, though no changes were made.

#### Impact of the parent meeting and MMR leaflet

The primary outcome measure was decisional conflict as measured by the Decisional Conflict Scale [42]. This generic measure is a 16-item scale to assess people's perceptions about the quality of their decision-making process; it has five sub-sections for being informed, clear about their values, degree of support, uncertainty with the choice, effectiveness of their decision. It has demonstrated test-retest reliability, construct and predictive validity in the patient health decision-making context [42]. Scores range from 1 (no decisional conflict) to 5 (extremely high decisional conflict). Scores lower than two are associated with 'implementing decisions', higher scores are interpreted as 'decision delay or feeling unsure about implementation'. High Cronbach alpha coefficients of 0.95 (T1), 0.92 (T2) and 0.94 (T3) were achieved.

Secondary outcomes were self-reported measures of the decision (actual and intended actions), attitude towards MMR and beliefs about the MMR options, knowledge and anxiety.

*The MMR decision *was measured at three months post-intervention using a self-report item 'Since this study started, have you taken your child to have the combined MMR vaccine?' In addition, a measure *of intended choice *was developed using three items measured on a 7-point scale e.g. 'I intend to give my child the combined MMR vaccine at the recommended ages' (definitely do not-definitely do) [43]. These three items were measured at all three time-points. Responses were averaged over the three items. Cronbach alpha coefficients of 0.84 (T1), 0.79 (T2) and 0.90 (T3) were obtained.

*Knowledge *about MMR and the measles disease was measured using multiple choice items developed for the purposes of this study using Department of Health for England and Wales literature [44]. The measure was not validated. The number of questions answered correctly were summed to produce a total knowledge score (maximum 11).

*Attitude towards MMR *was measured on a 7-point scale [43]. Parents responded to the statement 'For me to give my child the combined MMR vaccine at the recommended ages would be' on three semantic differential evaluative endpoints (1 to 7); e.g. extremely bad/extremely good. Responses were averaged over the three items. Cronbach alpha coefficients were 0.78 (T1), 0.73 (T2) and 0.80 (T3). These intended choice and attitude items have demonstrated validity and reliability and were informed by guidelines on measuring health cognitions [44].

*Parents' beliefs about the MMR options *were assessed using a modified version of the Beliefs about Flu Vaccination Questionnaire [45]. The measure was not validated for use in this context. Four items assessed parents' beliefs about the necessity of MMR e.g. 'Without the combined MMR vaccine, my child could get very ill from measles, mumps or rubella' and four items assessed parents' concerns about MMR e.g. 'Giving my child the combined MMR vaccine worries me'. All items were scored on a 5-point scale (strongly disagree-strongly agree). Items for each sub-scale were summed. Total scores for the two scales range from 4 to 20 with higher scores representing stronger beliefs in the necessity for, and concerns about, MMR. Cronbach alpha coefficients for the necessity sub-scale were 0.70 (T1), 0.63 (T2) and 0.70 (T3). Reliability was not improved by eliminating any items. Cronbach alpha coefficients for the concerns sub-scale were 0.77 (T1), 0.75 (T2) and 0.78 (T3).

*Anxiety *was measured to ensure that the parent meeting and MMR leaflet did not evoke anxiety in parents. We used the short form STAI [46]. Six items were used e.g. 'I feel calm', 'I am tense' and were scored on a 4-point scale (not at all-very much). The positive items (e.g. calm) were reverse scored and all six items were summed. The total score was multiplied by 20/6. A normal score is 34 to 36. High Cronbach alpha coefficients of 0.81 (T1), 0.86 (T2) and 0.84 (T3) were obtained.

### Sample size

To achieve 80% power to detect a standardised effect size of 0.67 on the primary outcome of decisional conflict [42,47], using a two-sided t-test with significance level of 0.05 and an estimated ICC of 0.05 (giving a design effect of 1.5 based on an average of 11 parents per cluster) required a sample size of 108 parents (54 in each group). Predicting 25% attrition 73 parents were required in each group. Parent numbers were not balanced across the clusters. Based on our previous research [9] we estimated recruiting 12 parents per week over three months.

### Analysis

An intention to treat analysis was conducted. This trial design was clustered within centres (healthcare centres, childcare organisations) and had repeated measures. The number of clusters and parents within each cluster were small in respect to multilevel modelling. To explore the potential effectiveness of the intervention on the primary outcome (decisional conflict) longitudinal analysis was used. This accounted for the multilevel structure of the data, with outcome measures collected at different time points within parent data. We were interested in exploring how decisional conflict changed over time with respect to covariates of interest, namely arm, focal MMR decision, parent characteristics (age, ethnicity, marital status, education, relationship to child, if have older child) and intended choice, knowledge, attitude, beliefs and anxiety at recruitment. A normal model was used, using MLwiN 2.10 beta 5 to perform these analyses.

Due to missing values, owing to non-completion of some questionnaire items, complete case analysis corresponded to only 65% of the data. Of these 92 parents, 44 (48%) were in the intervention arm and 48 (52%) were in the control arm. Missing values appeared to be at random. Multiple imputation was undertaken in Stata 10.0 to account for this. Five imputed datasets were generated using the results from linear regression analyses. Prior to undertaking multiple imputations seven parents were excluded (n = 3 intervention; n = 4 control) as they had not completed any study questionnaires, providing only parent characteristics data at recruitment. The best fit model for the complete case data was fitted to each imputed dataset and compared with the best fit model for those data. All imputed datasets agreed on the importance of the significant variables in the complete case model and results were found to be similar, thus indicating that minimal bias was introduced due to missing values. Aggregated results from the five imputed datasets using 135 participants (142 minus 7) are presented. Confidence intervals were calculated using the widest values to allow for errors generated through the imputation. Two sided significance tests and an alpha level of 0.05 were used throughout.

Repeated measures ANOVAs were computed for the secondary outcome measures using multilevel modelling using the aggregated results from the five imputed datasets. Chi squared analysis explored MMR uptake by arm.

## Results

### Intervention delivery

The parent meeting was delivered eight times during July and August 2006 in non-healthcare venues (e.g. community centres) close to participating healthcare centres and childcare organisations. Four daytime and four evening meetings were organised. Crèche facilities were provided at three daytime meetings. Forty one parents attended a parent meeting, 23 did not. Parents attending the meeting did not differ from those not attending the meeting in their characteristics or in their decisional conflict levels at baseline. The mean number of parents attending was 6 (range: 2 to 10). One meeting had less than four parents attending.

### Clusters and participants

**Figure 1:**
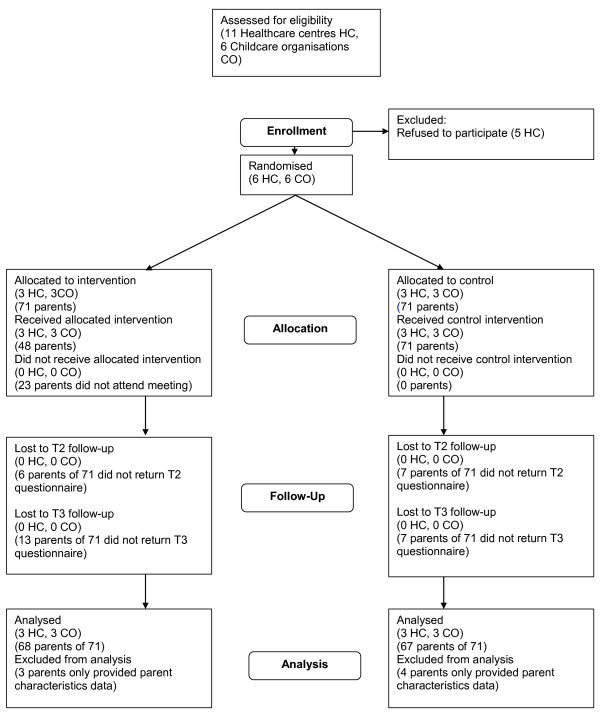
**Participant flow**.

Participant flow through the study is presented in Figure 1. The two arms were equivalent on all but one cluster characteristic. Mean list size for the childcare organisations was larger for the control arm (Table 2).

**Table 2:** Baseline characteristics of clusters and parents by arm

Characteristics	Intervention arm	Control arm
Primary healthcare centres, n	3	3

Childcare organisations, n	3	3

Mean healthcare centre parent list size	216	210

Mean childcare organisation parent list size	19	30

Mean Low Income Scheme Index score^a^	10	11

		

Parents, n	71	71

Mean age ± SD, yrs	34.07 ± 5.43	34.06 ± 5.52

Ethnicity, n (%)		
White British	68 (95.8%)	68 (95.8%)
Other	3 ( 4.2%)	3 ( 4.2%)

Marital status, n (%)		
Single or living with partner	27 (38.0%)	13 (18.3%)
Married or re-married	40 (56.4%)	57 (80.2%)
Separated/Divorced/Widowed	4 .(5.6%)	1 (1.5%)

Relationship to eligible child, n (%)		
Mother	67 (94.4%)	67 (94.4%)
Father	4 (5.6%)	4 (5.6%)

Education		
Left school at 16 years	24 (33.8%)	25 (35.2%)
Left school at 18 years	10 (14.1%)	10 (14.1%)
Achieved Degree or higher	37 (52.1%)	36 (50.7%)

Have older child		
Yes	36 (50.7%)	36 (50.7%)
No	35 (49.3%)	35 (49.3%)

First (youngest) child eligible, n (%) First dose MMR decision	23 (32.4%)	44 (62.0%)
Second dose MMR decision	48 (67.6%)	27 (38.0%)
Mean age ± SD of first (youngest) child eligible, months	25.73 ± 14.66	19.77 ± 11.69

Second youngest child eligible, n (%)		
First dose MMR decision	1 ( 4%)	0 (0.00%)
Second dose MMR decision	24 (96%)	22 (100.0%)

Mean age ± SD of second youngest child eligible, months	50.56 ± 17.13	49.32 ± 21.41

*Note*. N = 12 clusters, N = 142 parents. ^a^Low Income Scheme Index score is based on the percentage of prescribed items exempt from a prescription charge due to low income of the patient.

Of 1447 eligible parents invited, 150 (10%) consented to participate. Eight parents did not meet the inclusion criteria (did not have an 'actual' MMR decision to make at that time). Recruitment of 142 parents fell short of the 146 target allowing for 25% attrition, but the required sample size of 108 parents was achieved because of a less than anticipated drop out.

The two arms were equivalent on all parent characteristics (Table 2). There was a difference in the age of the first (youngest) eligible child and therefore in the MMR decision parents were making. One third of parents in the intervention arm were making a first dose decision compared with almost two thirds in the control arm. Dose decision was therefore modelled in the analysis.

### Impact of the parent meeting and MMR leaflet

#### Was the parent meeting associated with a reduction in decisional conflict?

**Figure 2:**
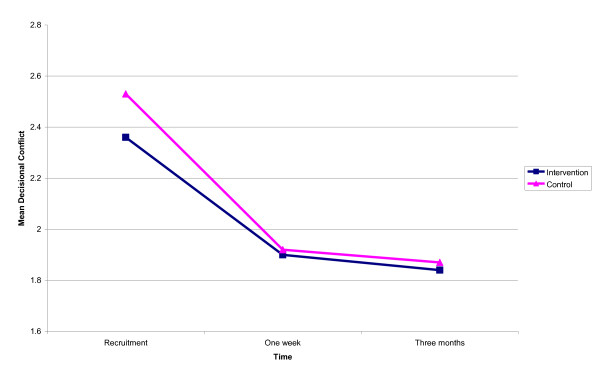
**Mean decisional conflict by arm over time**. Intervention/Control *Note*. Scores lower than two are associated with 'implementing decisions', higher scores are interpreted with decision delay or feeling unsure about implementation.

Mean decisional conflict by arm over time is presented in Figure 2. At recruitment parents in both arms reported levels of decisional conflict above two indicating that they had sufficient conflict about the choice to interfere with making the MMR decision effectively [42].

At one week post-intervention mean decisional conflict had decreased for both arms to below two; and remained below two at three months post-intervention. Time was significantly associated with decisional conflict. There was no significant association between arm and decisional conflict at any time point (see Table 3). In short, post-intervention, parents could implement an effective decision irrespective of arm allocation. The greatest reduction in decisional conflict occurred at one-week post-intervention. Focal MMR decision (first or second dose) was not significantly associated with decisional conflict i.e. perceived decisional conflict about this choice reduced over time for parents making first or second dose decisions.

**Table 3:** Coefficients for the longitudinal multilevel model of decisional conflict on potential covariates

Model variables	Effect Estimate	95% CI	p-value
Time-1 week post-intervention	-0.54	-0.67 to -0.41	**<0.001**

Time-3 months post-intervention	-0.60	-0.73 to -0.47	**<0.001**

Arm-intervention	0.07	-0.11 to 0.25	0.215

MMR decision-2^nd ^dose	-0.05	-0.24 to 0.14	0.310

Older child	-0.25	-0.42 to -0.07	**0.003**

Intended choice	0.09	0.02 to 0.17	**0.006**

Attitude	-0.20	-0.30 to -0.10	**<0.001**

Concern beliefs	0.07	0.04 to 0.10	**<0.001**

*Note*. N = 135. Results are presented per one point increase of decisional conflict.

#### Was the parent meeting associated with MMR decision?

Sixty six parents provided self-report data about their MMR choice. Of these 66, 29 (44%) were in the intervention arm and 37 (56%) were in the control arm. The remaining parents did not have children who were invited for vaccination within the study time period. Ninety three percent of parents in the intervention arm reported taking their child for the vaccination compared to 73% in the control arm. This difference was statistically significant (χ^2 ^(1, N = 66) = 4.43, 95% confidence interval 3.1% to 37.2%, p = 0.04).

#### Was the parent meeting associated with changes in parents' intended choice, knowledge, attitudes, beliefs or anxiety?

Time by group mean scores, 95% confidence intervals and significance levels for secondary outcomes are presented in Table 4. Small changes in the predicted direction were evident for the intervention arm for knowledge, intended choice, attitudes, and beliefs. However repeated measures ANOVAs revealed no significant time by arm effects. Mean anxiety remained below or within the normal range suggesting that neither the parent meeting nor the MMR leaflet evoked anxiety in parents.

**Table 4:** Descriptive data for secondary outcomes

		Intervention		Control		
**Outcome**	**Time point**	**Mean**	**95% CI**	**Mean**	**95% CI**	**p-value^a^**

Knowledge^b^	T1	6.38	6.00- 6.77	5.97	5.53- 6.41	0.253

	T2	8.22	7.84- 8.60	7.83	7.52- 8.15	

	T3	7.30	6.93- 7.66	7.08	6.80- 7.36	

Intended choice^c^	T1	5.93	5.54- 6.32	5.10	4.60- 5.60	0.605

	T2	6.03	5.67- 6.39	5.44	4.96- 5.92	

	T3	6.34	6.00- 6.68	5.58	5.06- 6.09	

Attitude^d^	T1	4.98	4.66- 5.30	4.52	4.17- 4.88	0.786

	T2	5.19	4.89- 5.48	4.77	4.44- 5.10	

	T3	5.26	4.96- 5.55	4.68	4.33- 5.04	

Necessity beliefs^e^	T1	16.88	16.22-17.53	16.73	15.99-17.47	0.578

	T2	17.63	16.97-18.28	17.18	16.49-17.87	

	T3	17.43	16.87-18.00	17.21	16.46-17.96	

Concern beliefs^e^	T1	9.83	8.92-10.73	11.00	10.04-11.96	0.939

	T2	8.92	8.15- 9.70	10.15	9.07-11.22	

	T3	8.66	7.81- 9.50	10.06	9.04-11.09	

Anxiety^f^	T1	32.50	29.63-35.37	34.20	31.52-36.89	0.219

	T2	30.89	28.00-33.77	33.78	30.43-37.13	

	T3	31.46	28.49-34.43	33.39	30.09-36.68	

*Note*. N = 135. ^a^Significance value for time by arm interaction. Values range from ^b^0 (no knowledge) to 11 (good knowledge); ^c^1(definitely do not intend) to 7 (definitely do intend); ^d^1 (extremely negative attitude) to 7 (extremely positive attitude); ^e^4 (not at all necessary/concerned) to 20 (very necessary/concerned); ^f^20 (low anxiety) to 80 (high anxiety).

### Which parent characteristics and cognitions were associated with changes in decisional conflict?

Baseline parent characteristics and outcome measures (irrespective of arm allocation) associated with changes in decisional conflict were: whether the parent had an older child; intended choice, attitude and concern beliefs (see Table 3).

If a parent had previously made an MMR decision for an older child, decisional conflict decreased over time by 0.25 compared to a parent who had not previously made an MMR decision.

Parents' concerns about the potiential adverse consequences of MMR at recruitment were significantly associated with changes in decisional conflict over time. The more concerned parents were, decisional conflict increased by 0.07.

For each additional point increase in attitude i.e. the more positive parents were about MMR, decisional conflict decreased by 0.20. For each additional point increase in intended choice, i.e. the stronger parents' intentions were to vaccinate, decisional conflict increased by 0.09.

## Discussion

In response to a continuing lack of confidence amongst many UK parents facing a decision about MMR [13-19] and informed by our earlier work [9,20] we developed and evaluated a parent-centred, multi-component intervention, delivered in community-based (non-healthcare) venues. We believe this to be the first study to evaluate a multi-component intervention to support informed decision making for MMR. Some study limitations should be acknowledged. Parent numbers were not balanced across the clusters thus reducing statistical power. However it seems unlikely that this would have changed the non-significant time by arm effect for decisional conflict. Only a small number of parents provided self-report data about their choice thus the study may have been under powered on this secondary outcome. The study was based in one city and only 10% of parents invited to take part did so. Due to the Data Protection Act [48] we cannot determine if they differ to non-responders. However the immunisation policy in Leeds mirrors UK policy and the sample was consistent with other MMR research that identifies parents who find it difficult to make this decision [9,13,22]. Finally, whilst complete case analysis was undertaken on just 65% of the data we believe that minimal bias was introduced.

The intervention was feasible to deliver in non-healthcare, community venues and it was acceptable to parents, with the majority expressing positive views [36]. Parents were equally positive about the MMR leaflet [36]. Our measure of decisional conflict showed a statistically significant decrease over time for both the intervention and control arms to a level where an informed decision for MMR could be made.

The positive effect of the MMR leaflet on decisional conflict observed in the control arm was unexpected. The inclusion of the leaflet was to reduce possible bias from a 'Hawthorne' effect [49]. Further, we considered the provision of a leaflet reflected usual vaccination practice. However, parents reported during meetings and in questionnaires that, contrary to stated local vaccination policy, leaflets were not routinely provided. For some parents this may have been because their child was not invited for MMR vaccination at the time of the study. Nevertheless, parents reported that this leaflet was more helpful in addressing their concerns about MMR compared to the usual Department of Health information at that time. Consequently instead of comparing our intervention with a control (usual care) we were comparing two different decision support interventions, a decision support leaflet versus participation in a parent meeting and a decision support leaflet. We were, therefore, unable to identify the independent effects on perceptions about the decision process of the parent meeting from usual care as originally intended.

This study did find that parents in the intervention arm were significantly more likely to report taking their child for the MMR vaccination than parents in the control arm. This suggests that providing information in a well-designed leaflet may be insufficient to lead to subsequent changes in the final choice (i.e. taking the child for immunisation). Enabling parents to act on their informed decisions may require a more pro-active approach than increasing knowledge and enabling clarification of their values [47]. In this study, it seems likely that the parent meetings provided sufficient decision support to enable parents to act on their decision, possibly illustrating a greater values-choice outcome. Parents making 'proxy' decisions about MMR on behalf of a child may require additional decision support where vaccination/non-vaccination consequences of regret and blame may be common and where media-induced controversy has adversely affected public trust in government and medical authorities [8,16]. In this vaccination context, the more proactive of the two decision support interventions was associated with more children receiving the MMR vaccination. We suggest that the concerns parents felt about this choice were met more fully by both interventions in this study than those parents who receive standard invitation letters for, and advice about, MMR in the UK [29].

Interestingly, for both groups the observed improvements in informed decision-making occurred for parents making first and second dose decisions. Previous research [9] would suggest that the first dose decision (for a first child) is the most anxiety-provoking and that parents may, therefore, experience greater uncertainty. Our study suggests that parents may benefit from concerted decision support for both doses. We also found that parents who had not previously made an MMR decision for an older child, those who were less positive in their attitude and more concerned about MMR had higher decisional conflict. These parents could usefully be targeted for decision support. Our finding that parents with strong intentions to vaccinate had higher decisional conflict suggests targeting parents who are approaching vaccination in the near future rather than those for whom vaccination and its potential consequences are more remote.

The majority of decision support research focuses on preference sensitive decisions [50] for which the 'best' decision is considered to be unclear and dependent on personal values. Immunisation is considered to be an 'effective' health decision [51] for which the weight of the scientific evidence would typically lead a health professional to recommend a particular course of action. This perhaps explains why governments and health professionals have historically adopted the 'knowledge deficit model' approach [52] of simply providing information and reassurance to parents. Irrespective of how childhood immunisation 'delivery systems' are organised [34,53], there is evidence from international literature [20] that parents' decision support needs are generally the same. Moreover, the below target MMR uptake rates in many countries suggests that reliance on a passive information-giving approach has limited effect. Comprehensive decision support, as provided in our parent meeting offers a potential solution.

## Conclusions

Whilst both the leaflet and the parent meeting reduced parents' decisional conflict, the parent meeting appeared to enable parents to act upon their decision leading to vaccination uptake. We are now testing the effectiveness of a web based MMR decision aid [27,54] for parents that could be made easily accessible, for example in public places where parents frequent such as schools, libraries, community centres as well as in the waiting rooms of healthcare centres.

## Competing interests

The authors declare that they have no competing interests.

## Authors' contributions

CJ and FMC conceived of the study, participated in its design and co-ordination, conducted the literature review and drafted the manuscript. RP ran the study and provided comments on the manuscript. HB and BL provided expert guidance on the design of the trial and provided comments on the manuscript. WH and RW conducted the statistical analysis and provided comments on the manuscript. All authors read and approved the final manuscript.

## Pre-publication history

The pre-publication history for this paper can be accessed here:

http://www.biomedcentral.com/1471-2458/11/475/prepub
